# Operating Room Nurses Want Differentiated Education for Perioperative Competencies—Based on the Clinical Ladder

**DOI:** 10.3390/ijerph181910290

**Published:** 2021-09-29

**Authors:** Yu Yeon Shin, Sang Suk Kim

**Affiliations:** 1 Department of Operating Room, Seoul National University Bundang Hospital, Seongnam-si 13620, Korea; ariel_1211@naver.com; 2 Red Cross College of Nursing, Chung-Ang University, Seoul 06974, Korea

**Keywords:** perioperative nursing, competence, clinical ladder

## Abstract

Operating room (OR) nurses’ perioperative competence is vital in operation and patient care. This cross-sectional descriptive study aimed to identify perioperative competencies and educational needs for improving competencies according to the clinical ladder. A total of 318 OR nurses in Korean tertiary hospitals were recruited. Data from the self-reported questionnaire of perioperative competencies, measured on a five-point Likert scale, were used. The average score of perioperative competence was 3.78 ± 0.54; among the sub-categories of competencies were collaboration (4.08 ± 0.55), foundational knowledge and skills (3.98 ± 0.56), proficiency (3.87 ± 0.64), empathy (3.77 ± 0.77), professional development (3.65 ± 0.64), and leadership (3.34 ± 0.89). There were significant differences in perioperative competencies according to the clinical ladder as follows: foundational knowledge and skills (*p* < 0.001), leadership (*p* < 0.001), collaboration (*p* = 0.017), proficiency (*p* < 0.001), and professional development (*p* < 0.001). The educational needs for foundational knowledge and skills (4.43 ± 0.60) were highest, and educational needs for proficiency (4.26 ± 0.70), collaboration (4.21 ± 0.77), leadership (4.08 ± 0.81), empathy (3.99 ± 0.91), and professional development (3.91 ± 0.76) were noted. The educational needs for improving perioperative competencies by clinical ladder showed a significant difference in leadership (*p* = 0.026), proficiency (*p* = 0.045), and professional development (*p* = 0.002). In order to develop an effective education program for OR nurses, differentiated education designs that reflect perioperative competencies and educational needs per clinical ladder are necessary.

## 1. Introduction

Nurses account for about 40% of the hospital workforce in Korea and play a crucial role in providing patient care and medical services [1,2]. The changing health care field in the Fourth Industrial Revolution requires advanced education that can continuously improve nursing professionalism and the competencies required of future nursing leaders [2]. Competency-based human resource management has the advantage of responding to shifts in human resource allocation or workforce development flexibly by focusing on the competencies that are commonly required for the position [3].

Operating room (OR) nurses participate in surgery teams with healthcare professionals such as surgeons, residents, anesthesiologists, anesthesia nurses, and radiologists. OR nurses must acquire knowledge related to surgical procedures, anesthesia, and patient’s medical conditions; adapt to the introduction of expensive high-tech equipment and complex new surgical techniques; and prevent patient safety accidents [4]. OR nurses must perform the tasks quickly and accurately by collaborating with healthcare professionals and exerting various competencies such as communication ability, leadership, and surgical knowledge and technology [5,6,7]. OR nurses’ perioperative competency is essential for coping with clinical situations, job involvement, job continuity, efficient operation management, and patient safety [8,9,10,11]. 

Recent studies in Korea regarding nursing competencies in the OR have focused on technologic tasks [4,12,13,14]. Most studies on the educational needs of perioperative competencies have been conducted for nursing students or new nurses, conjoining ORs and other units [15]. However, in the OR, non-technical aspects such as interpersonal relationships, communication skills, problem-solving skills, and critical thinking skills are also essential nursing skills [13,16,17]. Furthermore, nursing competency differs depending on clinical experience, and competency may differ even with similar experiences. In other words, the level of competency may vary depending on the level of experience and education [18]. In particular, OR nurses who perform various and complicated perioperative tasks require additional expertise as their responsibilities, experience, and role at work expand. Therefore, analyzing nursing competency, assessing the need for additional education that changes based on career stage and improves professionalism, and providing differentiated education according to the clinical ladder is very important for building capacity [5,13,18]. Additionally, education composition reflecting the clinical career of nurses can increase educational satisfaction and consequently strengthen perioperative competency [19]. 

This study aims to identify the perioperative competency of OR nurses according to the clinical ladder, including technical and non-technical aspects and educational needs for improving OR competency.

## 2. Materials and Methods

### 2.1. Design and Sampling

This study is a descriptive cross-sectional study to understand the perioperative competency level of OR nurses and the educational needs for improving perioperative competency. The participants were perioperative nurses who worked in the OR as scrub nurses, circulating nurses, educators, and managers. The sample pool was recruited using convenience sampling from general tertiary hospitals in Seoul and metropolitan areas of Korea. The number of samples for the study was calculated using the G*Power 3.1.9.2 program [20], with a significance level (α) of 0.05, an effect size (d) of 0.25, and a power (1-β) of 0.95 for an analysis of variance examining the differences among four clinical ladder groups. The OR nurses in this study were divided into four groups based on OR work experience and professionalism: ≤3, 4–6, 7–9, and ≥10 years. A sample of at least 280 participants was necessary according to the calculated results. In considering potential dropout (20%), 336 questionnaires were distributed between 28 August 2020 and 17 September 2020. Of this, 329 were returned; however, only 318 questionnaires were completed and analyzed (response rate: 94.6%).

### 2.2. Measures/Instruments

Using a self-reported online survey, we investigated the participants’ demographics, included age, sex, education status, clinical ladder, job position, experience as a preceptor, and job satisfaction. This study sought to analyze four groups of OR nurses based on the Dreyfus model of skill acquisition by Benner [21] and clinical career structure in a previous study [18]; the latter presented the four clinical ladders of Korean nurses divided by work experience and professionalism. 

The perioperative competence of OR nurses was measured using the Perceived Perioperative Competence Scale-Revised (PPCS-R). The PPCS-R was developed by Gillespie and Hamlin [22] and revised by Gillespie et al. [23]. The questionnaire was translated into Korean by researchers, and a bilingual professor confirmed reverse translation. The scale consists of 40 items across six domains: (1) foundational knowledge and skills (9 items), (2) leadership (8 items), (3) collaboration (6 items), (4) proficiency (6 items), (5) empathy (5 items), and (6) professional development (6 items). It is measured on a 5-point Likert scale from 1 (never) to 5 (always) on each item. Higher scores indicate greater levels of perceived competence. Cronbach’s α in the original study was 0.96. In the present study, Cronbach’s α was 0.96.

The education needed to improve perioperative care competence was measured using PPCS-R [23] with six domains: foundational knowledge and skills, leadership, collaboration, proficiency, empathy, and professional development. It was measured on a 5-point Likert scale from 1 (no need) to 5 (strong need). Higher scores indicate higher education is needed to improve competency. One open-ended question—“What education do you need to improve your perioperative competency?”—was added to identify additional information related to educational needs that reflect the work environment of a Korean OR. In the present study, Cronbach’s α was 0.97.

### 2.3. Data Analysis

Data were analyzed using IBM SPSS Statistics Version 22.0 (SPSS, Inc., Chicago, IL, USA). Descriptive statistics were reported in proportions or means and standard deviations (SD) for participants’ characteristics, perioperative competencies, and educational needs. The perioperative competencies and educational needs regarding demographic characteristics and clinical ladder were analyzed using a t-test, ANOVA, and post hoc analyses using Scheffe’s test.

### 2.4. Ethical Consideration

This study was approved by the institutional review board (IRB No. B-2005-613-301) of the researcher’s hospital.

## 3. Results

### 3.1. Difference in Perioperative Competencies and Educational Needs by Demographic Characteristics

The average age of the participants was 30.01 ± 6.18 years, and 276 were female (86.8%); 284 (89.3%) had bachelor’s degrees, 216 (67.9%) were staff nurses, 116 (36.5%) had experience as a preceptor, and 142 (44.6%) were satisfied with their job (Table 1). The participants’ average clinical experience in the OR was 81.82 ± 76.15 months; 117 (36.8%) had **≤** 3 years of experience, 73 (23.0%) had 4–6 years, 49 (15.4%) had 7–9 years, and 79 (24.8%) had ≥10 years. For the perioperative competencies, there were significant differences in age, education level, clinical ladder, job position, experience as a preceptor, and job satisfaction. Nurses of older age with a higher education level, a more senior position, and experience as a preceptor had higher competency scores (Table 1 and Table 2). For educational needs, there were significant differences in age and clinical ladder (Table 1 and Table 3).

### 3.2. Levels of Perioperative Competencies and Differences among Clinical Ladders

**Figure 1:**
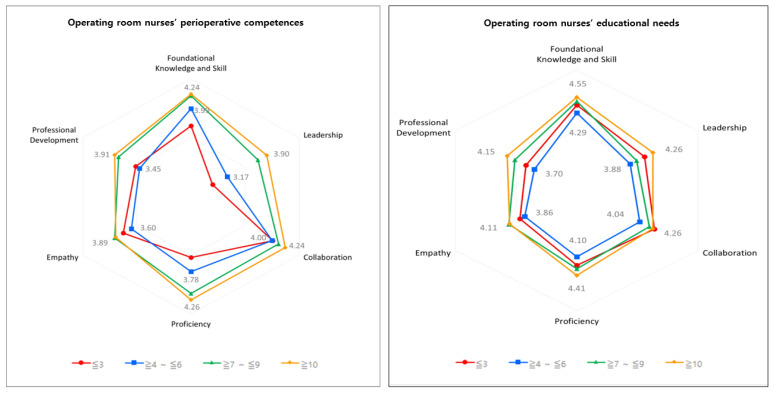
OR nurses’ perioperative competencies and educational needs.

The mean score for perioperative competencies was 3.78 ± 0.54. The score for collaboration was the highest at 4.08, followed by foundational knowledge and skills (3.98 ± 0.56), proficiency (3.87 ± 0.64), empathy (3.77 ± 0.77), professional development (3.65 ± 0.64), and leadership (3.34 ± 0.89). All competencies scored above average with 3.5 out of 5 points. Clinical career affected significant differences in most domains (*p* < 0.001)—specifically, foundational knowledge and skills, leadership, collaboration, proficiency, and professional development. The only competency that was not significantly different in the clinical ladder was empathy (Table 2; Figure 1).

### 3.3. Levels of Educational Needs and Differences among Clinical Ladders

The mean score and standard deviation for perioperative competencies was 4.22 ± 0.61. The score for foundational knowledge and skills was highest at 4.43 ± 0.60, followed by proficiency (4.26 ± 0.70), collaboration (4.21 ± 0.77), leadership (4.08 ± 0.81), empathy (3.99 ± 0.91), and professional development (3.91 ± 0.76). There were significant differences in educational needs according to clinical ladder, leadership, proficiency, and professional development. In contrast, the educational needs of foundational knowledge and skills, collaboration, and empathy were not significantly different in the clinical ladder (Table 3; Figure 1).

## 4. Discussion

This study identified that the OR nurses’ perioperative competencies and educational needs for improving perioperative competency differed across clinical ladder groups. In this study, the OR nurses’ perioperative competencies were highest in collaboration, followed by foundational knowledge and skills, proficiency, empathy, professional development, and leadership. Canadian and Australian OR nurses’ competencies were rated highly in empathy, foundational knowledge and skills, and collaboration, and low in leadership and professional development [24]. Further, in Istanbul, OR nurses’ collaboration and foundational knowledge and skills were high, and empathy and professional development were low [25]. However, Swedish OR nurses’ leadership, foundational knowledge and skills, and empathy were high, and collaboration and proficiency capabilities were low [26]. Cultural differences, hospital organizations, and differences in curriculum and professional course completion may have influenced the differences in perioperative competencies in each country [10]. However, foundational knowledge and skills were usually measured high, suggesting the importance of OR nurses’ knowledge and technical competency. In previous Korean studies, a direct comparison was limited as no studies used the same tools as the nursing perioperative competency tool used in this study. However, studies have reported strong confidence in foundational knowledge and skills to improve perioperative competency based on frequent work performance [27,28,29]. The relatively low leadership scores in this study were consistent with previous studies’ results. These results can be explained by many environmental factors [6,23,25], such as the urgent and busy work environment; the OR culture, which is focused on various complicated surgeries; and OR nurses’ perception that performing in a leadership role is challenging in the OR setting [27,28,29]. Therefore, it is a challenge to change the workplace system and OR nurses’ negative perceptions. However, considering the high educational need for leadership in the study, nurse educators and unit managers must provide strategies or programs for OR nurses to boost confidence and increase their leadership within the OR context.

For nurses’ perioperative competencies, according to the clinical ladder, the higher clinical experience group, especially those with seven to nine years and over 10 years of clinical experience, had higher perioperative competencies, which is consistent with the results of other studies [13,28]. Specifically, nurses with seven to nine years and over 10 years of clinical experience scored significantly higher in leadership, proficiency, and professional development than those with less clinical experience. This may be because in their roles as room charge or chief charge, nurses have many opportunities to improve technical competencies and non-technical perioperative competencies while educating nurses as preceptors, solving problems, and managing risk situations. Nurses with clinical careers of less than three years and between four and six years showed no significant difference in all domains. OR nurses with clinical careers of less than six years perform tasks similar to those of scrubs or circulating nurses, and they try to learn unexperienced surgeries according to medical part in turns. Further studies should focus on developing a clinical ladder system for OR nurses that reflects the OR system and work environment.

In this study, the educational needs for OR nurses’ perioperative competency improvement were highest in foundational knowledge and skills. Therefore, it is vital to maintain competitiveness and strengthen the competence of experts who adapt to the rapidly changing reality of the surgery field [30]. OR nurses should continuously participate in up-to-date practical education to achieve advanced technical skills and manage new equipment regardless of the clinical ladder. The educational needs of proficiency and collaboration were also high in this study. One explanation is that proficiency and collaboration are required to cope with crisis appropriately, reduce medical error, and ensure patient safety [4,31,32,33]. Furthermore, the high educational needs for leadership, professional development, collaboration, and empathy in this study suggest the necessity of education for technical and non-technical competencies in OR competency.

The educational needs differed significantly according to the clinical ladder in leadership, proficiency, and professional development. The educational needs of participants with over 10 years of clinical experience were significantly higher than those with four to six years of clinical experience. Nurses with more experience have specialized knowledge in perioperative nursing, make guidelines, train new nurses, solve problems, and offer job suggestions. Meanwhile, OR nurses with four to six years of clinical experience usually establish knowledge, skills, and attitudes about work and have confidence in their work [34]. However, they can fall into routines due to repetitive work and stress from increased responsibility in various work situations [35]. Considering the higher educational needs in the group with under three years of clinical experience compared to the four- to six-year group, this finding can be explained by educational needs being different depending on individual development efforts [13,28]. Therefore, internal and external rewards will help nurses with four to six years of clinical experience gain a sense of satisfaction or achievement and fully demonstrate their capabilities [36].

Consequently, securing high-quality human resources equipped with as much perioperative competency as possible helps to improve the quality of care and patient safety [37]. In developing educational programs for OR nurses, it is necessary to identify their competencies and reflect the need for continuous education to strengthen insufficient competencies according to their clinical experience and motivate the improvement of competencies [38].

## 5. Limitation

This study targeted nurses at tertiary hospitals in Seoul and metropolitan areas. Further, OR nurses in Korea alternate between a role as a scrub nurse and a circulation nurse when participating in operations; OR nurses with extensive experience also act as educators and managers. Due to these reasons, the study was limited in that the proportion of participants for each role could not be considered separately. Therefore, caution is advised regarding the generalizability of the study results. Due to a lack of a validated tool to measure perioperative competencies, a translated tool was used. Future research should focus on the development and validation of the instrument to incorporate considerations pertaining to the Korean organizational culture, hospital system, any cultural adaptations, and the competencies of OR nurses.

## 6. Conclusion

To improve OR nurses’ perioperative competencies, it is important to provide an effective, differentiated, and evidence-based educational program that reflects their educational needs, which differ according to the clinical ladder. This study found that the higher the clinical ladder, the higher the perioperative competencies. Moreover, OR nurses’ educational needs were high and differed according to the clinical ladder. These findings underline the necessity of differentiated educational content to reinforce the competency that is lacking depending on the clinical experience and the competency required for the next career stage when developing the program. This study may provide nurse educators and nurse leaders with ideas and material to design educational initiatives based on the specific educational needs in OR settings. Additionally, based on the findings of this study, future research could investigate the specific education content needed for each competency.

## Figures and Tables

**Table 1:** Difference in perioperative competencies and educational needs by demographic characteristics.

Variables	Classification	N (%) ^a^	Perioperative Competencies			Educational Needs		
			M ± SD	t or F	*p* (Scheffe)	M ± SD	t or F	*p* (Scheffe)
**Age**	<30 ^a^	196(61.6)	3.65 ± 0.51	18.265	<0.001 (a < b < c)	4.13 ± 0.67	4.601	0.011
	30–39 ^b^	84 (26.4)	3.90 ± 0.54			4.06 ± 0.65		(a < b< c)
	40≤ ^c^	38 (12.0)	4.15 ± 0.47			4.44 ± 0.56		
**Sex**	Male ^a^	276 (86.8)	3.79 ± 0.54	1.255	0.211	4.17 ± 0.65	3.553	0.060
	Female ^b^	42 (13.2)	3.68 ± 0.56			3.97 ± 0.74		
**Education level**	Bachlor’s degree ^a^	284 (89.3)	3.73 ± 0.54	−3.974	<0.001 (a < b)	4.13 ± 0.66	1.246	0.265
	Master’s degree ^b^	34 (10.7)	4.12 ± 0.44			4.27 ± 0.69		
**Job position**	Staff nurse ^a^ Room charge nurse ^b^ Chief charge nurse ^c^ Nurse educator ^d^ Unit manager ^e^	216 (67.9) 62 (19.6) 34 (10.7) 3 (0.9) 3 (0.9)	3.64 ± 0.50 3.94 ± 0.54 4.28 ± 0.39 4.26 ± 0.26 4.29 ± 0.17	16.226	<0.001 (a < e)	4.11 ± 0.68 4.07 ± 0.64 4.41 ± 0.56 4.45 ± 0.45 4.70 ± 0.20	2.372	0.052
**Experience as a preceptor**	Yes ^a^	116 (36.5)	3.91 ± 0.50	7.200	<0.001	4.15 ± 0.66	0.018	0.894
	No ^b^	202 (63.5)	3.72 ± 0.51			4.15 ± 0.66		
**Job satisfaction**	Very unsatisfied ^a^ Unsatisfied ^b^ Average ^c^ Satisfied ^d^ Very satisfied ^e^	12 (3.8) 31 (9.8) 133 (41.8) 125 (39.3) 17 (5.3)	3.88 ± 0.73 3.76 ± 0.70 3.64 ± 0.51 3.90 ± 0.48 3.83 ± 0.55	4.006	0.003 c < d	4.03 ± 0.94 4.16 ± 0.71 4.07 ± 0.66 4.20 ± 0.63 4.43 ± 0.56	1.509	0.199

Note: M: mean; SD: standard deviation; a, b, c, d, e, F: one-way analysis of variance and Scheffe multiple comparison analysis tests.

**Table 2:** Levels of perioperative competencies and differences among clinical ladders.

Competence	Subtotal	≤3 ^a^	≥4 ~ ≤6 ^b^	≥7 ~ ≤9 ^c^	≥10 ^d^	t or F	*p* (Scheffe)
		Mean ± SD	Mean ± SD	Mean ± SD	Mean ± SD		
Foundational knowledge and skills	3.98 ± 0.56	3.70 ± 0.53	3.99 ± 0.49	4.21 ± 0.58	4.24 ± 0.47	21.080	<0.001 (a < b< d)
Leadership	3.34 ± 0.89	2.90 ± 0.83	3.17 ± 0.80	3.74 ± 0.80	3.90 ± 0.71	30.200	<0.001 (a, b < c, d)
Collaboration	4.08 ± 0.55	4.01 ± 0.52	4.00 ± 0.55	4.12 ± 0.61	4.24 ± 0.53	3.442	0.017
Proficiency	3.87 ± 0.64	3.54 ± 0.57	3.78 ± 0.58	4.15 ± 0.64	4.26 ± 0.51	30.126	<0.001 (a, b < c, d)
Empathy	3.77 ± 0.77	3.75 ± 0.74	3.60 ± 0.74	3.91 ± 0.77	3.89 ± 0.80	2.508	0.059
Professional development	3.65 ± 0.64	3.52 ± 0.59	3.45 ± 0.59	3.84 ± 0.78	3.91 ± 0.56	10.482	<0.001 (a, b < c, d)
Total	3.78 ± 0.54	3.54 ± 0.46	3.67 ± 0.50	4.00 ± 0.58	4.08 ± 0.47	23.432	<0.001 (a, b < c, d)

SD: standard deviation; a, b, c, d, F: one-way analysis of variance and Scheffe multiple comparison analysis tests.

**Table 3:** Level of educational needs and differences among clinical ladders.

Competence	Subtotal	≤3 ^a^	≥4 ~ ≤6 ^b^	≥7 ~ ≤9 ^c^	≥10 ^d^	F	*p* (Scheffe)
		Mean ± SD	Mean ± SD	Mean ± SD	Mean ± SD		
Foundational knowledge and skills	4.43 ± 0.60	4.42 ± 0.60	4.29 ± 0.64	4.48 ± 0.60	4.55 ± 0.53	2.473	0.062
Leadership	4.08 ± 0.81	4.12 ± 0.79	3.88 ± 0.85	3.99 ± 0.93	4.26 ± 0.66	3.115	0.026 (b < d)
Collaboration	4.21 ± 0.77	4.28 ± 0.74	4.04 ± 0.82	4.20 ± 0.80	4.26 ± 0.72	1.609	0.187
Proficiency	4.26 ± 0.70	4.24 ± 0.68	4.10 ± 0.72	4.30 ± 0.76	4.41 ± 0.62	2.714	0.045
Empathy	3.99 ± 0.91	3.94 ± 0.93	3.86 ± 0.98	4.12 ± 0.95	4.11 ± 0.77	1.432	0.233
Professional development	3.91 ± 0.76	3.84 ± 0.74	3.70 ± 0.80	4.02 ± 0.80	4.15 ± 0.66	5.151	0.002 (b < d)
Total	4.22 ± 0.61	4.20 ± 0.61	4.07 ± 0.63	4.27 ± 0.67	4.35 ± 0.55	2.935	0.034 (b < d)

SD: standard deviation. a, b, c, d, F: one-way analysis of variance and Scheffe multiple comparison analysis tests.

## Data Availability

The data presented in this study are available on request from the corresponding author. The data are not publicly available due to restrictions, e.g., privacy or ethics.
